# Association between brain structures and migraine: A bidirectional Mendelian randomization study

**DOI:** 10.3389/fnins.2023.1148458

**Published:** 2023-03-03

**Authors:** Xiaoming Guo, Dingkun Wang, Caidi Ying, Yuan Hong

**Affiliations:** ^1^Department of Neurosurgery, The Second Affiliated Hospital, Zhejiang University School of Medicine, Hangzhou, China; ^2^Department of Neurosurgery, Tongde Hospital of Zhejiang Province, Hangzhou, China

**Keywords:** brain structure, NeuroImage, Mendelian randomization, migraine, cortex

## Abstract

**Background:**

Accumulating evidence of clinical and neuroimaging studies indicated that migraine is related to brain structural alterations. However, it is still not clear whether the associations of brain structural alterations with migraine are likely to be causal, or could be explained by reverse causality confounding.

**Methods:**

We carried on a bidirectional Mendelian randomization analysis in order to identify the causal relationship between brain structures and migraine risk. Summary-level data and independent variants used as instruments came from large genome-wide association studies of total surface area and average thickness of cortex (33,992 participants), gray matter volume (8,428 participants), white matter hyperintensities (50,970 participants), hippocampal volume (33,536 participants), and migraine (102,084 cases and 771,257 controls).

**Results:**

We identified suggestive associations of the decreased surface area (OR = 0.85; 95% CI, 0.75–0.96; *P* = 0.007), and decreased hippocampal volume (OR = 0.74; 95% CI, 0.55–1.00; *P* = 0.047) with higher migraine risk. We did not find any significant association of gray matter volume, cortical thickness, or white matter hyperintensities with migraine. No evidence supporting the significant association was found in the reverse MR analysis.

**Conclusion:**

We provided suggestive evidence that surface area and hippocampal volume are causally associated with migraine risk.

## Introduction

Migraine is a common and complex neurological disease associated with significant psychosocial impact, and has been a leading burden for global population health (Leonardi et al., 2005; Ashina et al., 2021). This disease is usually diagnosed on the basis of clinical criteria and can be further divided into two subtypes, including with- and without-aura (Charles, 2018; Ashina et al., 2021). In recent years, accumulating evidence of clinical and neuroimaging studies indicated that brain structural alterations played a pivotal role in migraine. These T1-weighted magnetic resonance imaging (MRI) studies used voxel-based morphometry or surface-based morphometry to explore brain morphology differences in volume, thickness or surface area (SA) between migraine patients and healthy controls (DaSilva et al., 2007; Schmidt-Wilcke et al., 2008; Datta et al., 2011; Messina et al., 2013; Rocca et al., 2014; Coppola et al., 2017; Husøy et al., 2019; Amin et al., 2021; Kim et al., 2021). Currently, migraine has been hypothesized to be a both neuronal and vascular genetic disorder upon the mainstream opinion (Schwedt and Dodick, 2009; MacGregor, 2017). The latest and largest genome-wide association study (GWAS) meta-analysis, including 102,084 migraine cases and 771,257 controls, identified 123 risk loci associated with migraine, which were enriched in both vascular and central nervous system tissue/cell types (Hautakangas et al., 2022). And it supported the concept that migraine is a neurovascular disorder (Hautakangas et al., 2022).

Previous studies have revealed that cortex in migraine patients has peculiar anatomical, functional, and neurochemical properties (Barbanti et al., 2019). And the incidence of white matter hyperintensities obviously increased in migraine patients (Eikermann-Haerter and Huang, 2021). Hippocampus plays an important role in the processing of pain, pain-related attention, and stress response (Liu et al., 2018). Besides, the volume of hippocampus has been found to be related to migraine prognosis (Liu et al., 2018). Although the significant relationships between brain morphology alterations and migraine could be observed in previous studies, it remains unclear whether brain morphology alterations are the cause or consequence of migraine attacks. The relationship between hippocampal volume and migraines remains unclear, with some studies suggesting a positive correlation and others a negative one. Thus, it is necessary to clarify the direction of the association and provide evidence at the genetic level between brain structures and migraine.

Mendelian randomization (MR) is a genetic epidemiologic method by using genetic variants associated with exposures, which can avoid many of the potential methodological limitations of observational studies, such as reverse causation bias and confounding (Smith and Ebrahim, 2003). In view of the basis that both brain morphometry and migraine risk are influenced by genetic factors, using MR analysis to improve the knowledge of the relationship between brain morphometry alterations and migraine is promising. Recently, only one MR study have investigated the association between intracranial volume and migraine (Mitchell et al., 2022). Important brain traits like cortical SA, cortical thickness, and some other traits of interests were not assessed. Here, we carried out an MR study to explore the causal association between these important brain traits and migraine risk using the largest GWAS data.

## Materials and methods

### Study design

**FIGURE 1:**
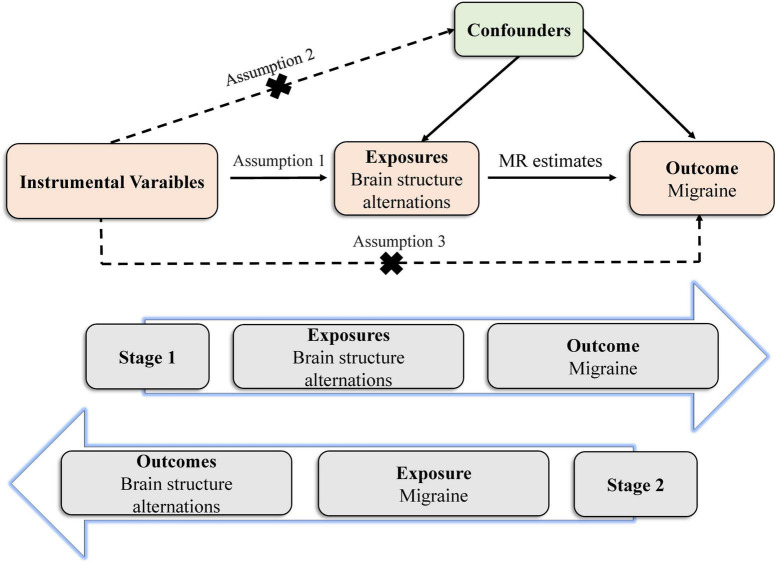
Principles of the Mendelian randomization study for brain structural alterations and migraine risks.

The MR study builds on three predominant assumptions (Figure 1). (1) Selected instrumental variables (IVs) are strongly and consistently associated with exposures. (2) There is no association between the IVs and confounders. (3) IVs impact outcomes through exposures directly, but not other pathways. Genetic variants are frequently utilized as IVs, due to their well-defined nature and resistance to alteration by environmental factors, thereby avoiding reverse causation (Burgess et al., 2017b). We conducted this bidirectional MR study to clarify the causal relationship between brain structures and migraine.

### Genetic instruments selection and data sources

Genetic variants associated with total cortical SA and average cortical thickness were obtained from a genome-wide meta-analysis, which were based on 51,665 predominantly healthy participants in the Enhancing NeuroImaging Genetics through Meta-Analysis Consortium and the UK Biobank. The total SA and average cortical thickness were measured on MRI (Grasby et al., 2020). A total of 12 SNPs associated with total SA and 2 SNPs associated with average cortical thickness were collected (*P* ≤ 8.3 × 10^–10^). For gray matter volume (GMV), we drew 8 SNPs (*P* ≤ 5 × 10^–8^) from a large GWAS of brain imaging-derived phenotypes from an open web server (the Oxford Brain Imaging Genetics),^1^ which included 33,224 participants from the UK Biobank (Smith et al., 2021). As for white matter hyperintensities (WMH), we obtained 24 SNPs as IVs of WMH from 50,970 individuals from Cohorts for Heart and Aging Research in Genomic Epidemiology consortium and from the UK Biobank (*P* < 5 × 10^–8^) (Sargurupremraj et al., 2020). The aggregated risk variants were then confirmed to be associated with WMH in another cohort of 1,738 young healthy adults (*P* = 2.5 × 10^–7^), which provided insight into the lifetime impact of WMH. In addition, we extracted six independent SNPs as IVs significantly associated with hippocampal volume (HV) from a genome-wide meta-analysis with 33,536 individuals (*P* ≤ 5 × 10^–8^), which accounted for as much as 18.76% of the variance in HV (Hibar et al., 2017). All IVs included were clumped for independence (*r*^2^ < 0.1; region size, 3000 kb) according to the Europeans data from the 1,000 Genomes Project. If these included SNPs were not available in the outcome datasets, proxy SNPs (*r*^2^ > 0.8) were acquired online as replacements.^2^

The summary-level data of migraine was obtained from the largest available genome-wide meta-analysis, combining five migraine study collections and comprising of 102,084 migraine cases and 771,257 controls of European ancestry (Hautakangas et al., 2022). A total of 123 independent SNPs associated with migraine were utilized as IVs in reverse analysis (*P* < 5 × 10^–8^). In this study, migraine phenotype was defined by self-reported information or second edition of international Classification of Headache Disorders. Logistic regression analyses were conducted by adjusting for age, sex, and at least for the four ancestry principal components. We also used a web tool^3^ to estimate the bias due to sample overlap and the calculated results were negligible (<1%). All participants gave written informed consent in these studies, and sites involved obtained approval from local research ethics committees or Institutional Review Boards.

### Statistical analysis

In the main analyses, we applied the random-effects and fixed-effects inverse-variance weighted (IVW) approach to obtain causal estimates (Burgess et al., 2017a). We conducted several sensitivity analyses to identify potential pleiotropy. Cochran’s Q test was used to evaluate the heterogeneity among different instrumental variables (Burgess et al., 2017a). Weighted median method allowed less than 50% of the genetic variants to be invalid instrumental variables (Burgess et al., 2018). MR-Egger method was conducted to detect and adjust pleiotropic bias (Burgess and Thompson, 2017). To further control potential pleiotropy, we used the MR Pleiotropy Residual Sum and Outlier (MR-PRESSO) method to conduct a global test of heterogeneity and identify horizontal pleiotropy (Verbanck et al., 2018). Once the pleiotropic outlier instruments were identified, a repeated IVW analysis after removing these outlier instruments would be performed (Verbanck et al., 2018).

All tests were two sided and the Bonferroni-corrected significance threshold was set to *P* < 0.005 (correcting for 10 outcomes). The *P*-values between 0.005 and 0.05 was defined as suggestive of potential association between exposure and outcome. Odds ratios (ORs) are presented for each 1 standard deviation difference in all exposures. All analyses were conducted by using TwoSampleMR and MR-PRESSO packages in R software (Version 4.1.3).

## Results

The main characteristics of datasets adopted in the MR analyses were shown in Supplementary Table 1. All F-statistics of these IVs were higher than the threshold of 10, suggesting no weak instrument bias in the present study. The summary information of SNPs on the five traits was shown in Supplementary Table 2.

**FIGURE 2:**
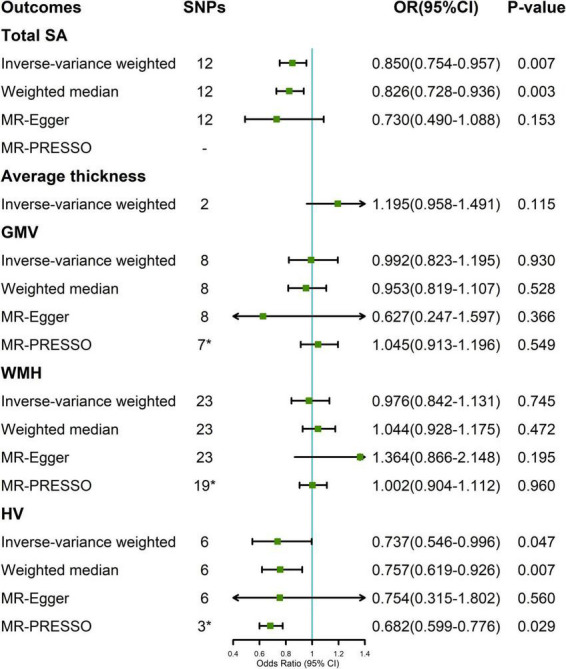
Association between genetically determined brain structural alterations and migraine risks. *MR-PRESSO outlier detected (Supplementary Table 2). CI, confidence interval; OR, odds ratio; SNP, single nucleotide polymorphism; WMH, white matter hyperintensities.

In the random-effect IVW estimates, genetically increased cortical SA was potentially associated with a decreased risk of migraine (OR = 0.850; 95% CI, 0.754–0.957; *P* = 0.007; Figure 2). This association was robust in the weighted median and MR-Egger. And MR-PRESSO did not identify any potential SNP outliers and we did not observe evidence of horizontal pleiotropy in MR-Egger (P for intercept = 0.452). The Cochran’s Q test indicated significant heterogeneity (Cochran Q-derived *P* = 0.032). There was no significant evidence for association of cortical thickness with migraine (OR = 1.195; 95% CI, 0.958–1.491; *P* = 0.115).

A suggestive association was found between genetically determined HV and migraine (OR = 0.737; 95% CI, 0.546–0.996; *P* = 0.047; Figure 2). The Cochran’s Q test indicated significant heterogeneity (Cochran Q-derived *P* < 0.001). There was no indication of horizontal pleiotropy in the association of HV with migraine as measured by MR-Egger (P for intercept = 0.959). Three SNPs (rs11979341, rs61921502, and rs7020341) were detected in the MR-PRESSO test, and the result remained suggestive (*P* = 0.029). Genetically determined GMV and WMH were not causally associated with migraine in the IVW method. The lack of causality was also confirmed in the weighted median, MR-Egger estimates, and MR-PRESSO.

There were no any significant effects of migraine on brain structural alternation in the reverse estimates (Supplementary Table 3).

## Discussion

This MR analysis revealed a suggestive causal association of genetically decreased SA and HV with higher risk of migraine. In addition, there was no evidence supporting the association between genetic liability to migraine and brain structural alterations in the reverse MR analysis.

The literatures on the association between brain structural alterations and migraine remained inconclusive. Several observational studies demonstrated that the global or regional SA decreased in migraine cases compared with healthy controls (Messina et al., 2013; Petrusic et al., 2018; Planchuelo-Gómez et al., 2020). And it frequently involved the visual motion processing, pain processing, and executive function regions (Messina et al., 2013; Planchuelo-Gómez et al., 2020). Cortical SA is usually thought to be congenital and is largely independent of environmental or external factors (Kapellou et al., 2006; Frye et al., 2010; Messina et al., 2013). Some authors supposed that cortical SA could be a good biomarker to distinguish migraine patients from healthy controls (Petrusic et al., 2018). Our results of bidirectional MR analysis further supported the causal association between cortical SA and migraine risk. However, the causal relationship was only found in total SA instead of regional SA due to low variance explained by SNPs. Thus, further studies are required to identify the association between migraine and the related regional SA.

Cortical thickness is a marker reflecting gray matter integrity, which can be determined by the number of their neurogenic divisions. In previous literatures, variation in regional or global cortex thickness was controversial (DaSilva et al., 2007; Messina et al., 2013; Husøy et al., 2019; Torres-Ferrus et al., 2021). A possible explanation was that the sample of included subjects was rather small, while the traits to be compared were many, which might lead to false positive results in this way of comparison. Besides, cortical thickness might undergo considerable changes postnatally. For patients with migraine, several factors might affect cortical thickness, such as age, disease duration, frequency of the attacks, and even the scanning timing (attack phase vs. interictal phase). Some authors hypothesized that increased cortical thickness was a compensatory mechanism to meet the requirement for increased sensory processing of migraine attacks. Similar results of increased cortical thickness were found in other neuropsychiatric disorders, including schizophrenia, autism spectrum disorder, early-stage Parkinson’s disease and so on (Biundo et al., 2013; Williams et al., 2022). Although the direction of the association in our analysis was in accordance with previous studies, the result was not significant (*P* = 0.115). In addition, only two available SNPs were applied as IVs in the causal estimate, which might influence the result.

Besides, the decrease in HV was suggestively associated with an increased risk of migraine, which was in lined with most of previous studies (Liu et al., 2013, 2017; Chong et al., 2017). At the same time, observational evidence showed that HV had been negatively associated with the frequency and severity of migraine attacks (Maleki et al., 2013; Liu et al., 2017). Mitchell et al. (2022) suggested that migraine attacks might influence HV in a longitudinal study. However, this reverse relationship was not verified in our study or a recent MR study. WMH and GMV were reported to be associated with migraine. Some studies suggested that a patent foramen ovale might be associated with migraines, accompanied by changes in the gray matter and destruction of the white matter (Signoriello et al., 2018; Cao et al., 2022). Possible mechanisms implicated in the pathophysiology of this phenomenon include microembolus-triggered cortical spreading depression, the vasoactive substance hypothesis, impaired cerebral autoregulation, and a common genetic basis (Signoriello et al., 2018; Cao et al., 2022). However, these association were not discovered in our bidirectional MR analysis. In fact, null findings in our study do not truly reflect lack of associations, since MR analyses are dependent on the power of the original GWAS to a large extent (Pierce and Burgess, 2013).

There are some strengths in our study. Firstly, the use of bidirectional MR design permitted an examination of the reverse causation. Secondly, several sensitivity analyses, including pleiotropy robust methods were applied to ensure the valid estimation of the causal effect size. In addition, we used well-powered GWAS data of migraine, which had more statistical power to detect associations than past smaller study. Finally, our results were in lined with a recent MR study based on voxel-based morphometry, which additionally increased the robustness of our results (Mitchell et al., 2022).

This study also has some limitations. Firstly, we only used total cortical SA and average thickness to explore the causal association between cortical structure and migraine, since the fraction of variance explained by SNPs was low in regional SA and thickness. Secondly, sample overlapping may occur between exposure and outcomes population, especially for cohort of UK biobank, which may potentially bias the results. But recently, one study showed that two-sample MR can be applied safely and robustly in a single large dataset using large biobanks (Minelli et al., 2021). Thirdly, most of participants in this study were of European descents, which limited our findings to extend to other ancestries.

In conclusion, we provided suggestive evidence that decreased cortical SA, and decreased HV are suggestively associated with higher migraine risk, and we did not find any significant effect of migraine on brain structural alternation in the reverse estimates. Future investigation into the brain regions is recommended, which helps to clarify the underlying mechanisms and point to new therapies against migraine.

## Data availability statement

The original contributions presented in this study are included in the article/Supplementary material, further inquiries can be directed to the corresponding author.

## Ethics statement

Ethical review and approval was not required for the study on human participants in accordance with the local legislation and institutional requirements. Written informed consent for participation was not required for this study in accordance with the national legislation and the institutional requirements.

## Author contributions

XG: drafting/revision of the manuscript, study design, and analysis and interpretation of data. DW: analysis and interpretation of data. CY: acquisition of data. YH: revision of the manuscript. All authors contributed to the article and approved the submitted version.

## Footnotes

1
https://open.win.ox.ac.uk/ukbiobank/big40/

2
https://ldlink.nci.nih.gov/

3
https://sb452.shinyapps.io/overlap/
