# Supplementary material for: Association between brain structures and migraine: A bidirectional Mendelian randomization study
Source: Front Neurosci. 2023 Mar 3;17:1148458. doi: 10.3389/fnins.2023.1148458 (PMC10020331; doi:10.3389/fnins.2023.1148458)
Supplement: Supplementary file 1 [file Table_1.DOCX]

| Tratis | Data source | Sample size(cases/controls) | Ancestry |
| --- | --- | --- | --- |
| Migraine | IHGC2016, UKBB, deCODE, DBDS, LUMINA | 102,084 cases/771,257 controls | Europeans |
| Surface area and thickness | ENIGMA, UKBB | 33,992 | Europeans |
| Grey matter volume | UKBB | 8,428 | Europeans |
| White matter hyperintensities | CHARGE, UKBB | 50,970 | 48,454 Europeans and 2,516 African-Americans |
| Hippocampal volume | ENIGMA, CHARGE | 33,536 | Europeans |

eTable 1. Studies and datasets adopted in the MR analyses.

eTable 2. Summary information of the single nucleotide polymorphisms used as instrumental variables for five traits.

| Traits | SNP | EA | OA | EAF | Beta | SE | P | Sample size | R2 | F-statistics | Outlies in MRPRESSO |
| --- | --- | --- | --- | --- | --- | --- | --- | --- | --- | --- | --- |
| Total Surface Area | rs12630663 | T | C | 0.5883 | -0.03835 | 0.00674 | 1.27E-08 | 32176 | 0.000712 | 22.93655 |  |
| Total Surface Area | rs34464850 | C | G | 0.1534 | 0.074732 | 0.009255 | 6.76E-16 | 31984 | 0.001451 | 46.46029 |  |
| Total Surface Area | rs2301718 | A | G | 0.2269 | 0.044676 | 0.008021 | 2.55E-08 | 32176 | 0.0007 | 22.54555 |  |
| Total Surface Area | rs7715167 | T | C | 0.3857 | -0.04016 | 0.00722 | 2.65E-08 | 32068 | 0.000764 | 24.53 |  |
| Total Surface Area | rs2802295 | A | G | 0.3793 | -0.0433 | 0.006847 | 2.54E-10 | 32176 | 0.000883 | 28.43458 |  |
| Total Surface Area | rs11154343 | T | C | 0.3183 | -0.04728 | 0.007478 | 2.59E-10 | 31547 | 0.00097 | 30.62547 |  |
| Total Surface Area | rs11759026 | A | G | 0.7624 | -0.07887 | 0.008158 | 4.11E-22 | 31907 | 0.002254 | 72.07016 |  |
| Total Surface Area | rs12357321 | A | G | 0.3206 | -0.04234 | 0.007251 | 5.22E-09 | 32176 | 0.000781 | 25.15108 |  |
| Total Surface Area | rs1628768 | T | C | 0.7614 | -0.05896 | 0.008 | 1.70E-13 | 32176 | 0.001263 | 40.69388 |  |
| Total Surface Area | rs11171739 | T | C | 0.5721 | -0.04219 | 0.006874 | 8.41E-10 | 31319 | 0.000871 | 27.31371 |  |
| Total Surface Area | rs10878349 | A | G | 0.49 | 0.063024 | 0.006696 | 4.83E-21 | 32176 | 0.001985 | 64.00027 |  |
| Total Surface Area | rs79600142 | T | C | 0.7802 | 0.102829 | 0.008682 | 2.33E-32 | 29435 | 0.003627 | 107.1288 |  |
| Average Thickness | rs11692435 | A | G | 0.091 | -0.08273 | 0.013636 | 3.18E-10 | 29128 | 0.001132 | 33.01456 |  |
| Average Thickness | rs533577 | T | C | 0.4935 | -0.04545 | 0.007273 | 8.43E-11 | 32872 | 0.001033 | 33.98598 |  |
| Grey matter volume | rs10445367 | T | G | 0.18 | -0.06 | 0.01 | 9.30E-09 | 8428 | 0.001063 | 8.964005 | Yes |
| Grey matter volume | rs10520585 | G | C | 0.24 | 0.053 | 0.0093 | 9.60E-09 | 8428 | 0.001025 | 8.643175 |  |
| Grey matter volume | rs11867479 | T | C | 0.36 | -0.053 | 0.0081 | 5.90E-11 | 8428 | 0.001294 | 10.92064 |  |
| Grey matter volume | rs1485302 | A | G | 0.43 | -0.047 | 0.0078 | 2.20E-09 | 8428 | 0.001083 | 9.134 |  |
| Grey matter volume | rs2536185 | T | G | 0.45 | 0.077 | 0.0078 | 5.70E-23 | 8428 | 0.002935 | 24.80188 |  |
| Grey matter volume | rs3205187 | C | G | 0.66 | -0.046 | 0.0082 | 2.40E-08 | 8428 | 0.00095 | 8.009448 |  |
| Grey matter volume | rs9788296 | T | C | 0.49 | -0.05 | 0.0078 | 2.00E-10 | 8428 | 0.00125 | 10.54146 |  |
| Grey matter volume | rs9843908 | C | T | 0.56 | 0.042 | 0.0078 | 4.90E-08 | 8428 | 0.000869 | 7.331088 |  |
| WMH | rs10786772 | G | A | 0.64 | 0.042 | 0.006 | 1.56E-12 | 50970 | 0.000813 | 41.4631 |  |
| WMH | rs11257311 | G | T | 0.7 | 0.046 | 0.01 | 2.01E-08 | 50970 | 0.000889 | 45.33657 |  |
| WMH | rs12443113 | G | A | 0.55 | 0.031 | 0.006 | 3.42E-08 | 50970 | 0.000476 | 24.25676 |  |
| WMH | rs1285847 | T | C | 0.55 | 0.036 | 0.006 | 1.24E-10 | 50970 | 0.000642 | 32.71798 |  |
| WMH | rs12921170 | A | G | 0.58 | 0.05 | 0.006 | 9.82E-18 | 50970 | 0.001218 | 62.15473 |  |
| WMH | rs17205972 | T | G | 0.2 | 0.049 | 0.007 | 4.76E-12 | 50970 | 0.000768 | 39.18984 |  |
| WMH | rs1948948 | C | T | 0.56 | 0.037 | 0.006 | 1.17E-10 | 50970 | 0.000675 | 34.40843 |  |
| WMH | rs2303655 | T | C | 0.78 | 0.048 | 0.007 | 4.03E-11 | 50970 | 0.000791 | 40.33396 | Yes |
| WMH | rs34974290 | A | G | 0.19 | 0.104 | 0.007 | 2.59E-46 | 50970 | 0.003329 | 170.2477 |  |
| WMH | rs4630220 | G | A | 0.71 | 0.048 | 0.007 | 1.46E-13 | 50970 | 0.000949 | 48.40371 |  |
| WMH | rs55940034 | G | A | 0.29 | 0.037 | 0.006 | 4.49E-09 | 50970 | 0.000564 | 28.74963 | Yes |
| WMH | rs5762197 | C | A | 0.71 | 0.039 | 0.007 | 2.67E-08 | 50970 | 0.000626 | 31.9437 |  |
| WMH | rs62172472 | G | A | 0.79 | 0.047 | 0.007 | 3.67E-11 | 50970 | 0.000733 | 37.3842 |  |
| WMH | rs6503417 | C | T | 0.63 | 0.052 | 0.006 | 3.43E-19 | 50970 | 0.001261 | 64.3316 |  |
| WMH | rs6540873 | A | C | 0.61 | 0.027 | 0.007 | 1.37E-08 | 50970 | 0.000347 | 17.6848 |  |
| WMH | rs6797002 | C | T | 0.73 | 0.049 | 0.007 | 8.18E-14 | 50970 | 0.000946 | 48.2856 |  |
| WMH | rs6940540 | G | T | 0.41 | 0.045 | 0.006 | 7.17E-15 | 50970 | 0.00098 | 49.98206 |  |
| WMH | rs71471298 | T | C | 0.11 | 0.053 | 0.009 | 2.72E-09 | 50970 | 0.00055 | 28.04794 | Yes |
| WMH | rs7157599 | C | T | 0.29 | 0.041 | 0.007 | 1.49E-08 | 50970 | 0.000692 | 35.30631 |  |
| WMH | rs73184312 | G | A | 0.72 | 0.038 | 0.006 | 2.18E-09 | 50970 | 0.000582 | 29.69192 |  |
| WMH | rs73923006 | G | C | 0.81 | 0.055 | 0.007 | 2.00E-14 | 50970 | 0.000931 | 47.50028 |  |
| WMH | rs7596872 | A | C | 0.1 | 0.097 | 0.01 | 3.81E-24 | 50970 | 0.001694 | 86.46687 |  |
| WMH | rs7603972 | A | G | 0.87 | 0.07 | 0.01 | 2.23E-13 | 50970 | 0.001108 | 56.5546 | Yes |
| Hippocampal volume | rs77956314 | T | C | 0.916 | -0.1252 | 0.012 | 2.06 E-25 | 26814 | 0.002412 | 64.83231 |  |
| Hippocampal volume | rs61921502 | T | G | 0.8466 | 0.0834 | 0.0093 | 1.94E-19 | 26814 | 0.001807 | 48.52664 | Yes |
| Hippocampal volume | rs11979341 | C | G | 0.6837 | -0.0485 | 0.0072 | 1.42E-11 | 24484 | 0.001017 | 24.93258 | Yes |
| Hippocampal volume | rs7020341 | C | G | 0.359 | 0.046208 | 0.007 | 3.04E-11 | 26700 | 0.000983 | 26.26206 | Yes |
| Hippocampal volume | rs2268894 | T | C | 0.5412 | -0.04382 | 0.007 | 5.89E-11 | 26814 | 0.000954 | 25.59419 |  |
| Hippocampal volume | rs2289881 | T | G | 0.3544 | -0.03877 | 0.007 | 2.73E-08 | 26814 | 0.000688 | 18.45127 |  |

EA = effect allele; EAF = effect allele frequency; OA = other allele; SE = standard error; SNP = single nucleotide polymorphisms, WMH = white matter hyperintensities.

eTable3 Reverse estimates of migraine on brain structural alternations

| Outcomes | OR (95% CI) | P | SNPs |
| --- | --- | --- | --- |
| Total Surface Area | 0.984(0.945-1.025) | 0.448 | 107 |
| Average thickness | 0.990(0.951-1.031) | 0.637 | 107 |
| Grey matter volume | 1.024(0.980-1.070) | 0.289 | 116 |
| White matter hyperintensities | 1.054(0.995-1.116) | 0.074 | 116 |
| Hippocampal volume | 0.987(0.936-1.041) | 0.631 | 115 |
